# External Locus of Control but not Self-Esteem Predicts Increasing Social Anxiety Among Bullied Children

**DOI:** 10.32872/cpe.3809

**Published:** 2022-06-30

**Authors:** Belinda Graham, Lucy Bowes, Anke Ehlers

**Affiliations:** 1 Department of Experimental Psychology, University of Oxford, Oxford, United Kingdom; 2 Oxford Health NHS Foundation Trust, Oxford, United Kingdom; Philipps-University of Marburg, Marburg, Germany

**Keywords:** ALSPAC, bullying, social anxiety, locus of control

## Abstract

**Background:**

Elevated social anxiety is more likely among bullied children than those who have not been bullied but it is not inevitable and may be influenced by cognitive factors. Lower self-esteem and more external locus of control are associated with bullying and social anxiety but the impact of these factors over time among bullied children is less clear.

**Method:**

Children from the UK Avon Longitudinal Study of Parents and Children (ALSPAC) reported bullying experiences at age 8 (n = 6,704) and were categorized according to level of bullying exposure. The impact of self-esteem and locus of control on social anxiety was assessed up to age 13 across the bullying exposure groups using multi-group latent growth curve analysis. Complete data was available for 3,333 participants.

**Results:**

More external locus of control was associated with a steeper increase in social anxiety among severely bullied children [B = .249, p = .025]. Although self-esteem at age 8 was associated with existing social anxiety it did not predict later increases in social anxiety.

**Conclusion:**

These results indicate that beliefs about lack of personal control among severely bullied children may contribute to increasing social anxiety over time. Exploring related cognitions may be helpful in this potentially vulnerable group.

## Highlights

- Childhood bullying increases risk of social anxiety.
- For severely bullied children this risk is exacerbated by external locus of control.
- Exploring beliefs around lack of personal control may be helpful.

Social anxiety is characterised by excessive fears of coming across badly or being judged harshly by others in social situations (American Psychiatric Association, 2013; World Health Organisation, 1992) and can lead to avoidance and poorer performance in school, work, and relationships (e.g., Stein & Kean, 2000; Van Ameringen et al., 2003). It is a chronic treatable condition (Bruce et al., 2005) that is maintained by unhelpful cognitions (Clark & Wells, 1995). Childhood bullying increases the risk of developing social anxiety (Arseneault, 2018; Pontillo et al., 2019) with higher risk conferred by more frequent exposure (Copeland et al., 2013) but not all bullied children are socially anxious and identifying subgroups at risk may inform prevention and intervention. Previous cross-sectional research has identified locus of control (Reknes et al., 2019) and self-esteem (Wu et al., 2021) as modifiers of the relationship between bullying and mental health outcomes. In this longitudinal study, we evaluate the impact of locus of control and self-esteem on social anxiety over time among children with different levels of bullying exposure. Better understanding factors that contribute to the unfolding of social anxiety symptoms in young people over time could inform targeted and developmentally appropriate approaches to treatment.

Bullying is generally understood to include aggressive interpersonal acts that are intentional, repeated, and include a power imbalance between the victim and the aggressor (Olweus, 1994). Prevalence rates vary according to the measure of bullying used, setting and child age. One survey found rates of 8.7-14.4% for frequent bullying and 26.8-38.1% for occasional bullying over a 10-year period in England (Chester et al., 2015). Bullying experiences are classified as *overt* events like hitting, threatening or name calling, and *relational* events that use social power to inflict hurt by excluding, ignoring, gossiping or telling lies behind someone’s back. Experiences like these can be socially traumatic (Wild & Clark, 2011) and contribute to the onset and maintenance of anxiety disorders (Norton & Abbott, 2017) including social anxiety (Hackmann et al., 2000). Of note, problematic social anxiety commonly arises during adolescence, with rates of onset peaking around age 13 (Kessler et al., 2005). Among adults with anxiety disorders, those suffering with social anxiety are particularly likely to report having been bullied or teased when they were younger (McCabe et al., 2003, 2010). Increased risk of elevated long-term anxiety after bullying is evident from retrospective studies (Gladstone et al., 2006) and prospective data (Copeland et al., 2013; Gladstone et al., 2006; Sourander et al., 2007; Stapinski et al., 2014). Therefore, it is well established that bullying increases risk of social anxiety. However, mechanisms are less well understood.

Locus of control (Nowicki & Duke, 1974) refers to the extent to which someone believes the outcomes of events or behaviours to be under personal control (internal) or down to luck or chance (external). Internal locus of control is associated with better wellbeing while external locus of control is associated with negative outcomes such as depression (Zhang et al., 2014) and higher levels of PTSD, for example among survivors of combat (Karstoft et al., 2015) and children exposed to stressful political life events (Hallis & Slone, 1999). It is possible that these outcomes are driven by associations with thinking and coping styles, such that internality is associated with positive thinking and help-seeking, while externality is associated with avoidance and helplessness (Reknes et al., 2019). Research has shown that adolescents who are victims of bullying generally have a more external locus of control compared with peers not involved in bullying (Radliff et al., 2016) and among severely bullied adolescents those with more external locus of control also had higher risk of psychotic symptoms (Fisher et al., 2013). Of note, Reknes et al. (2019) suggested that externality may contribute to a diminished sense of personal responsibility that is actually protective for adult victims of workplace bullying, as they may more readily attribute negative experiences externally. This may suggest a reduced risk of negative outcomes for bullied children who have a more external locus of control. However, no longitudinal studies have specifically investigated locus of control as a mechanism driving social anxiety among bullied children.

Low self-esteem refers to an unfavourable attitude towards the self (Rosenberg, 1979) and may be informed by negative social interactions including experiences of bullying that are internalised (van Geel et al., 2018). Cross-sectional studies show that lower self-esteem is associated with bullying (Brito & Marluce, 2013; O’Moore & Kirkham, 2001) and cyberbullying (Patchin & Hinduja, 2010) but cannot speak to the direction of the effect, such that although bullying may contribute to reducing self-esteem it is also possible that children with lower self-esteem are more likely to be targeted (van Geel et al., 2018). Wu et al. (2021) found that self-esteem explained some of the cross-sectional relationship between bullying and social anxiety among adolescents, but did not investigate causation due to the study design. In this study we investigate longitudinally whether lower self-esteem increases the risk of social anxiety among children who are bullied.

Cognitive models suggest that negative beliefs maintain social anxiety (Clark & Wells, 1995) and PTSD (Ehlers & Clark, 2000). For social anxiety, beliefs are commonly connected in meaning to past experiences of humiliation or rejection (Wild et al., 2007) and include themes of personal capacity to perform adequately and appear acceptable to other people (e.g., “*I am inadequate*”). For PTSD, beliefs are commonly connected with the traumatic event and its sequelae and include themes about loss of control in terms of personal reactions (e.g., “*I cannot handle stress*”) and the environment more broadly (e.g., “*The world is completely dangerous*”). Of note, these maintaining cognitions related to self and past or future events are not limited to explicit thoughts, but rather include imagery and “felt sense” that is highly emotional (Ehlers et al., 2004; Hackmann et al., 2000). Beliefs consistent with external locus of control and low self-esteem may be fruitful targets for cognitive interventions with bullied children if these factors negatively impact anxiety trajectories in this group.

The current study assessed the moderating effects of locus of control and self-esteem on social anxiety among children using a three-wave longitudinal design over five-years, from age 7.5 to 13. The goal of this study was to assess whether externality of locus of control and self-esteem at age 8 influence the trajectory of social anxiety among children up to the age of 13, and whether the impact of these cognitive factors differs depending on bullying exposure. Therefore, this study hypothesized that (1) social anxiety will increase from age 7.5 to 13; (2) children exposed to more severe peer victimisation will have higher initial social anxiety and steeper increase in social anxiety over time; (3) lower self-esteem, and (4) more external locus of control will predict higher initial social anxiety and steeper increase over time for those with more severe victimization experiences.

## Method

**Figure 1:**
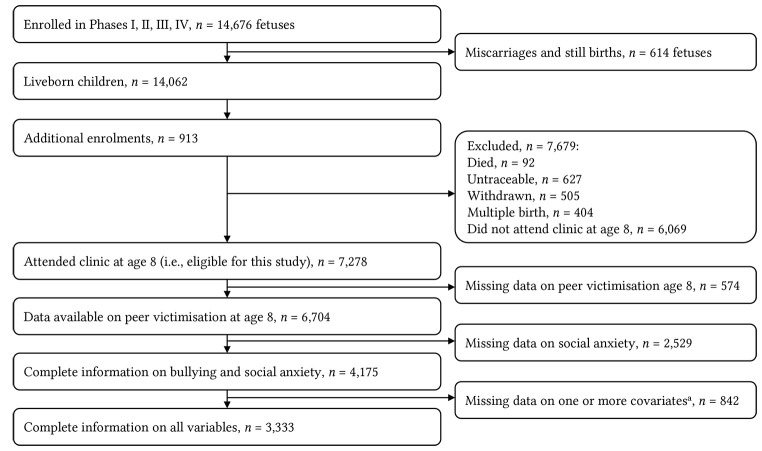
Participant Flowchart ^a^Prior emotional problems (Strengths and Difficulties Questionnaire), locus of control (Nowicki-Strickland Internal-External Scale), self-esteem (Harter’s Self-Perception Profile for Children). Data available at www.bristol.ac.uk/alspac/researchers/cohort-profile/

This sample was drawn from the Avon Longitudinal Study of Parents and Children (ALSPAC) which is a large prospective observational study of health and development in children. Pregnant women resident in Avon, UK during 1991-2 were invited to take part in the study. Of 14,541 pregnancies initially enrolled, there was a total of 14,676 foetuses, resulting in 14,062 live births and 13,988 children who were alive at 1 year of age. When the oldest children were approximately 7 years of age, an attempt was made to bolster the initial sample with eligible cases who had failed to join the study originally. The number of new pregnancies not in the initial sample (known as Phase I enrolment) is 913 (456, 262 and 195 recruited during Phases II, III and IV respectively). The phases of enrolment are described in more detail in the cohort profile paper and its update (Boyd et al., 2013; Fraser et al., 2013). The total sample size is therefore 15,454 pregnancies, resulting in 15,589 foetuses, of whom 14,901 were alive at 1 year of age. This includes multiple births. Participant flowchart shown in Figure 1. Informed consent for the use of data collected via questionnaires and clinics was obtained from participants following the recommendations of the ALSPAC Ethics and Law Committee at the time. Please note that the study website contains details of all the data that is available through a fully searchable data dictionary and variable search tool (http://www.bristol.ac.uk/alspac/researchers/our-data/). This project proposal received approval from ALSPAC executive committee [B2804].

### Participants

In total 7,278 participants attended clinic assessment at age 8 making them eligible for this study. Of these, 6,704 provided data on bullying exposure, of whom 2,529 were missing data on social anxiety at one or more time points and 842 were missing data on one or more covariates. Complete data was therefore available for 3,333 cases. The current sample includes singleton births only to reduce within family confounds.

### Measures

#### Peer Victimisation

A modified version of the bullying and friendship interview (Wolke et al., 2001) was used to determine the frequency that children had experienced nine different types of relational and overt peer victimisation involving other children at school or to/from school in the past six months. Specifically, four relational behaviours (others wouldn’t play with them to upset them, been made to do things didn’t want to do, had lies/nasty things said about them, had games spoilt) and five overt behaviours (had personal belongings taken, been threatened/blackmailed, been hit/beaten up, been tricked in a nasty way, been called bad/nasty names). For each type, participants responded “*no*”, “*yes sometimes*” (less than four times), “*yes repeatedly*” (four or more times), or “*yes very frequently*” (at least once per week). Fisher et al. (2013) established an index of bullying severity in the same sample comprising three levels of bullying severity at age 8, such that children who reported exposure to both overt and relational victimization at least 4 times each or at least once per week were classed as severely bullied, those who had experienced only one of these types at this frequency were classed as occasionally bullied, and all remaining children were classified as not bullied. Internal reliability was acceptable (α = 0.73).

#### Locus of Control

An adapted version of the Nowicki-Strickland Internal-External scale (Nowicki & Duke, 1974) suitable for use with children was completed during in-person assessment at age 8 years, comprising 12 items answered yes/no. A sum score was calculated (Range 0-12), with higher scores indicating more external locus of control and lower scores indicating more internal locus of control.

#### Self-Esteem

The global self-worth subscale of Harter’s Self Perception Profile for Children (Harter, 1985) was completed during in-person assessments at age 8 years, comprising 6 items each split into two components reflecting high and low self-esteem (e.g., some children are often unhappy with themselves, other children are pretty pleased with themselves). Each component was rated as “*sort of true for me*” or “*really true for me*” to produce a four-point scale for each item. A sum score was calculated (Range 6 – 24), with higher scores indicating higher self-esteem. Internal reliability was acceptable (α = 0.73).

#### Prior Emotional Problems

Parents rated their child’s emotional wellbeing using the relevant subscale from the Strengths and Difficulties Questionnaire at age 6.75 years. A sum score was calculated (Range 0 – 10) with higher scores indicating more emotional difficulties. This variable was included as a covariate in the model.

#### Social Anxiety

Parents rated their child’s fear of new people, lots of people, and eating, speaking, reading, or writing in front of others over the last month as either “*no*”, “*a little*”, “*a lot*”, using the Development and Well-being Assessment (Goodman et al., 2000) six-item social fears subscale (DAWBA-SF) at age 7.5, 10, and 13. A total score was calculated (Range 0 – 12), with higher scores indicating more severe social anxiety. Internal reliability was good (α = 0.77 – 0.80).

### Analytic Approach

First, the pattern of growth in social anxiety over time was modelled using a first-order latent growth curve model (LGCM), specifying initial severity (intercept) and shape of change (slope) using a repeated measure sum score of severity of social anxiety (DAWBA-SF). Data was collected at three time points so linear shape was assumed and loadings for time were fixed at 0 (baseline, age 7.5), 2 (age 10), and 5 (age 13) in order to allow interpretation of the intercept as severity at age 7.5 and slope as linear change over time (Hypothesis 1). Intercepts and slopes were allowed to vary between individuals. Good model fit was assessed using recommended indices (Hooper et al., 2008), namely standardized root mean square residual (SRMR) below 0.08, root mean square error of approximation (RMSEA) below 0.05, comparative fit index (CFI) above 0.95, and Tucker-Lewis Index (TLI) above 0.90. Models were run in Mplus using the MLR estimator (maximum likelihood estimation with robust standard errors) to minimize bias associated with missing data from study attrition and to account for non-normality of observations. Chi-square significance was not used to assess model fit as it is unreliable in large samples and is not estimated when using MLR. All measures were assumed to be influenced by random measurement error.

Second, to test the hypothesis that trajectory of social anxiety differs by level of exposure to victimisation (Hypothesis 2), exposure to victimisation was tested as a predictor of social anxiety overall, and in addition initial level and slope was compared between not bullied (*n* = 4,037), occasionally bullied (*n* = 1,955), and severely bullied (*n* = 712) groups in a multi-group LGCM grouped by exposure to victimisation. Presence of additional variance in social anxiety trajectory was also assessed within each victimisation exposure group, with and without adjustment for prior emotional problems.

**Figure 2:**
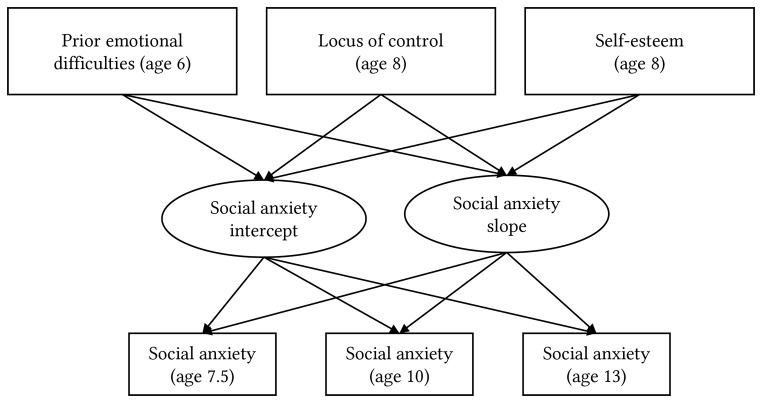
Diagram of Multigroup Latent Growth Curve Model, Grouped by Bullying Severity (“Not”, “Occasionally”, “Severely”)

Third, to test the contribution of cognitive predictors (Hypothesis 3, 4) locus of control and self-esteem were entered into the model to determine their ability to explain variance in initial level and slope in the full sample, in each bullying exposure group, and between bullying exposure groups (see Figure 2).

Univariate Anova suggested no evidence of a differential relationship between bullying and social anxiety according to sex so analyses were conducted on the group as a whole. Data was inspected in SPSS27 and analysed in Mplus 8.

## Results

### Sample Characteristics

Enrollment and participation flowchart is shown in Figure 1. At age 8, over a third of participants (*n* = 2,667, 39.8%) reported exposure to either relational or overt victimization at least four times over the last six months, and of these over a quarter (*n* = 712, 26.7%) experienced both types and were classified as severely bullied.

Severity of bullying exposure at age 8 was not associated with level of social anxiety but was associated with lower self-esteem and more external locus of control. Those exposed to bullying also had higher prior emotional difficulties compared to those not exposed to bullying. Characteristics are shown in Table 1.

**Table 1:** Sample Characteristics by Severity of Bullying Victimisation at Age 8

Variable	Severity of Bullying at age 8, *M* (*SD*) or *n* (%)			
	Not bullied (*n* = 4,037)	Occasional (*n* = 1,955)	Severe (*n* = 712)	*F*, χ^2^
Gender Female (*n*, %)	2,136 (52.9)	910 (46.5)	341 (47.9)	17.68^a^
Social Anxiety				
Age 7.5	0.83 (1.50)	0.81 (1.44)	0.86 (1.67)	0.31 (ns)
Age 10	0.89 (1.57)	0.92 (1.62)	1.01 (1.76)	1.49 (ns)
Age 13	1.12 (1.75)	1.17 (1.80)	1.32 (1.97)	3.23^a^
Self-Esteem	19.67 (3.18)	18.98 (3.51)	18.02 (3.72)	80.14^b^
Locus of Control	5.71 (2.05)	6.21 (2.00)	6.67 (2.12)	78.69^b^
Prior emotional difficulties	1.43 (1.58)	1.57 (1.74)	1.59 (1.71)	5.06^c^

*Note.* Self-reported severity of bullying victimisation using Bullying and Friendship Interview and categorized following Fisher et al. (2013); Social anxiety = DAWBA Social Fears subscale (Range 0 – 12; higher is more social anxiety); Self-esteem = Harter’s Self Perception Profile for Children: Shortened Form (Range 6 – 24; higher is better self-esteem); External locus of control = Nowicki-Strickland Internal External Scale (Range 0 – 12; higher is more external); Prior emotional difficulties = relevant subscale from Strengths and Difficulties Questionnaire age 6.75 (Range 0 – 10; higher is more emotional difficulties). Significant group difference between, a. “*not bullied*” and “*severe*”, *p* < .05, b. all groups, *p* < .01, c. “*not bullied*” and both bullied groups (*p* < .05).

### Missing Data

Among the sample with data on bullying at age 8, missing data on social anxiety at age 13 was more likely among those who were severely bullied, χ^2^(2, *n* = 6,704) = 9.89, *p* = .007 and those whose parents had a lower socio-economic status, χ^2^(1, *n* = 5,601) = 29.84, *p* < .001. Missingness did not differ by sex, χ^2^(1, *n* = 6,704) = 0.241, *ns*.

### Trajectory of Social Anxiety

A single linear growth curve model of social anxiety over time had a good fit for the data, CFI = .992, TLI = .977, SRMR = .010, RMSEA = .037, 90% CI [0.018, 0.060]. Across the sample, the social anxiety variable was highly positively skewed but mean levels increased slightly from age 7.5 (Range 0 – 12, *M* = 0.83, *SD* = 1.51), to age 10 (Range 0 – 12, *M* = 0.91, *SD* = 1.60), to age 13 (Range 0 – 11, *M* = 1.15, *SD* = 1.79), confirmed by small but significant positive slope (*M* = 0.07, *SE* = .005, *p* < .001). There was also significant variability in social anxiety intercept (*M* = 1.27, *SE* = .090, *p* < .001) and slope (*M* = 0.55, *SE* = .008, *p* < .001) indicating individual differences around the mean trajectory.

Model fit improved when level of prior emotional difficulties, which are expected to be associated with social anxiety at age 7.5, was included in the model, CFI = .996, TLI = .987, SRMR = .009, RMSEA = .024, 90% CI [0.010, 0.040]. Prior emotional difficulties predicted initial social anxiety (*M* = 0.39, *SE* = .021, *p* < .001) but did not impact on the rate of subsequent change in social anxiety over time (*M* = -0.02, *SE* = .030, *ns*).

### Trajectories of Social Anxiety by Severity of Bullying Exposure

When severity of bullying exposure at age 8 was added as a predictor of overall social anxiety trajectory alongside prior emotional problems, the expected effect of bullying on social anxiety was shown, such that higher bullying exposure at age 8 predicted a slightly steeper increase in social anxiety over time (*M* = 0.06, *SE* = 0.02, *p* = .014). However, bullying exposure at age 8 was not associated with concurrent social anxiety (*M* = -0.01, *SE* = 0.02, *ns*).

**Figure 3:**
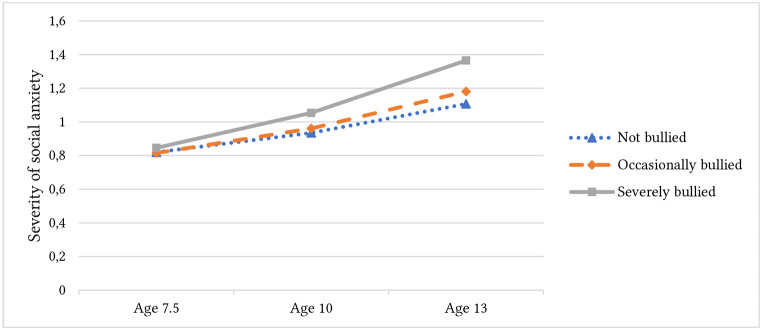
Estimated Mean Social Anxiety (Age 7.5 – 13) Grouped by Bullying Exposure at Age 8 *Note.* Grouped by self-reported level of bullying victimisation in Bullying and Friendship Interview. Social anxiety assessed at three time points (age 7.5, 10, 13) using DAWBA Social Fears subscale (Range 0 – 12; higher is more social anxiety). At age 10, group differences in social anxiety are not significant. At age 13, social anxiety was significantly higher in the severe group compared with the not bullied group (*p* = .013) but not the occasionally bullied group (*p* = .090).

In order to test the impact of cognitive factors on social anxiety in the context of differing bullying exposure, the sample was split following Fisher et al. (2013) into three groups of bullying severity, namely “*not bullied*”, “*occasional*”, and “*severe*”. Model fit for this grouped model was good, CFI = .995, TLI = .986, SRMR = .011, RMSEA = .025, 90% CI [0.007, 0.042]. All three groups had significant positive slope indicating increasing social anxiety over time (“*not bullied*”: *M* = .063, *SE* = 0.01, *p* < .001; “*occasional*”: *M* = .075, *SE* = .01, *p* < .001; “*severe*”: *M* = .102, *SE* = .02, *p* < .001) but Hypothesis 2 was not supported as there were no significant differences in mean initial social anxiety severity or mean slope between bullying exposure groups. See Figure 3. Of note, there was significant variance in slope within each group indicating that other factors are responsible for explaining individual differences in trajectory.

### Cognitive Predictors of Social Anxiety by Severity of Bullying Exposure

The final model including hypothesized predictors (locus of control, self-esteem, and prior emotional difficulties) had good model fit, CFI = .995, TLI = .986, SRMR = .010, RMSEA = .019, 90% CI [0.004, 0.032]. Contrary to Hypothesis 3, lower self-esteem at age 8 was not independently associated with concurrent social anxiety or rate of change in social anxiety over time in any group. In contrast, while more external locus of control at age 8 was not associated with concurrent social anxiety in any group, it predicted a moderate increase in anxiety in the severely bullied group (*B* = .167, *p* = .011) with smaller effects for those who were never bullied (*B* = .095, *p =* .005) and occasionally bullied (*B* = .097, *ns*). Prior emotional problems strongly predicted social anxiety at age 7.5 in all groups but did not impact the rate of change in social anxiety over time. See Table 2.

**Table 2:** Predictors of Social Anxiety Trajectory From Age 7.5 to 13 Grouped by Age 8 Bullying Exposure

Trajectory components and predictors	Not bullied, *n* = 4,037		Occasional, *n* = 1,955		Severe, *n* = 712	
	Coefficient (*SE*)	*p*	Coefficient (*SE*)	*p*	Coefficient (*SE*)	*p*
**Intercept (social anxiety age 7.5)**	**0.816 (.024)**		**0.810 (.035)**		**0.846 (.066)**	
Predictors of intercept						
Self-Esteem	-0.041 (.023)	*ns*	-0.059 (.034)	*ns*	0.009 (.055)	*ns*
Locus of control	0.039 (.024)	*ns*	0.038 (.034)	*ns*	0.075 (.049)	*ns*
Prior emotional problems	0.393 (.026)	< .001	0.412 (.043)	< .001	0.344 (.063)	< .001
Slope (social anxiety over time)	**0.057 (.007)**		**0.073 (.010)**		**0.102 (.018)**	
Predictors of slope						
Self-Esteem	-0.021 (.033)	*ns*	0.027 (.048)	*ns*	-0.031 (.069)	*ns*
Locus of control	0.095 (.034)	< .05	0.097 (.051)	*ns*	0.167 (.065)	< .05
Prior emotional problems	-0.033 (.040)	*ns*	-0.013 (.057)	*ns*	-0.008 (.081)	*ns*
Intercept x Slope	**-0.061 (.023)**		**-0.067 (.035)**		**-0.081 (.070)**	

*Note.* Cells contain unstandardized coefficients for intercept and slope estimated without predictors, standardized coefficients for predictors, with standard errors (*SE*) and probabilities (*p*; two-tailed). Social Anxiety = DAWBA Social Fears subscale; Self-esteem = Harter’s Self Perception Profile for Children: Shortened Form; Locus of control = Nowicki-Strickland Internal External Scale; Prior emotional problems = mother report relevant subscale from Strengths and Difficulties Questionnaire.

## Discussion

Children who were bullied at age 8 were more likely to have a more external locus of control than other children and higher externality among severely bullied children was associated with steeper increases in social anxiety up to the age of 13. Of note, exposure to bullying at age 8 was not associated with existing social anxiety at age 7.5 but was associated with subsequent increases social anxiety, and this increase was larger for those with external locus of control. This pattern was not observed in relation to self-esteem. This suggests that external locus of control in early childhood could be a risk factor for later anxiety among severely bullied children and is a potential target for intervention.

External locus of control describes a tendency to consider events and experiences as outside personal control. In this sample, the effect of externality on negative outcomes was small overall but larger in the severely bullied group, such that external locus of control was associated with steeper increases in social anxiety for severely bullied children. Beliefs around bullying that are consistent with external locus of control may include thoughts such as, “*being picked on is inevitable*”, or “*others will always target me*”. Evidence from personal experiences that contradict these types of beliefs related to bullying may be accessible for those who are not bullied or bullied occasionally. In contrast, beliefs about lack of control over a threatening and unpredictable social environment may be strengthened by repeated confirmatory evidence for children who are severely bullied and therefore more likely to persist. In line with existing literature suggesting that external locus of control is a risk factor for psychopathology (Hallis & Slone, 1999; Karstoft et al., 2015; Zhang et al., 2014), there was some indication of a dose response relationship between external locus of control and social anxiety, but with only minimal effects among children who were never or occasionally bullied. Reknes et al. (2019) suggested that external beliefs were protective against general psychological strain for adult victims of workplace bullying, enhancing acceptance and enabling external attribution of negative experiences towards negative characteristics of the perpetrator or bad luck instead of taking personal blame. However, the current study suggests that while external control beliefs do not confer additional risk of social anxiety for occasionally bullied children there is an additional risk for severely bullied children. For children who are severely bullied and have a tendency towards externality, promoting personal control beliefs may be one route towards encouraging more constructive coping strategies.

The cognitive model of social anxiety disorder (Clark & Wells, 1995) posits that those suffering with social anxiety hold unhelpful beliefs about their ability to perform well in social situations that, when triggered in a social situation, lead to increased self-consciousness and self-monitoring. In an effort to mitigate the perceived risks, the person then engages in “safety-seeking” behaviors that are intended to keep them safe (e.g. looking down and avoiding eye contact). However, these behaviors can also have unintended consequences (e.g. looking unfriendly or disinterested) which negatively impact the social interaction. Therefore, it is possible that excessive perception of threat may persist even in the absence of an ongoing objectively threatening environment and that perceiving threat may encourage children to act in ways that could inadvertently increase likelihood of ongoing bullying. In this analysis, social anxiety at age 7.5 was not associated with higher bullying exposure at age 8, so it is not necessarily the case that more fearful children were being targeted or perceived that they were being targeted. However, appraisals associated with external locus of control may contribute to excessive perceptions of ongoing social threat and to passive or unhelpful forms of coping that contribute to increasing social anxiety over time.

It is interesting to note that early self-esteem did not influence the trajectory of social anxiety to age 13 among any bullying exposure group. As such, it is possible that early cognitive processes related to self-esteem could be less important in terms of predicting future anxiety (Sowislo & Orth, 2013) despite evidence of cooccurrence (Lee & Hankin, 2009). In fact, these results support cross-sectional associations between bullying, self-esteem and social anxiety (Gómez-Ortiz et al., 2018; Núñez et al., 2021) but our findings suggest that children who are bullied and have low self-esteem are not necessarily at increased risk of social anxiety over time. Similarly, although our analyses showed the expected association between prior emotional problems and social anxiety at age 7.5, there was no ongoing impact of early emotional problems on increasing social anxiety over time. This indicates that early emotional problems and self-esteem may be less important indicators of ongoing adjustment compared with external locus of control, a feature that has been largely overlooked in this domain but which may be an important clinical target for assessment and intervention.

This study has important clinical implications. It is notable that long-term anxiety associated with bullying can persist even in the absence of current threat, that is, even after bullying has stopped. Cognitive theories of anxiety after stressful experiences (Clark & Wells, 1995; Ehlers & Clark, 2000) suggest that cycles develop between unhelpful beliefs, particular memory characteristics, and behavioral and cognitive coping strategies to maintain anxiety. Maladaptive beliefs associated with bullying may contribute to social anxiety that increases over time and children who have been bullied may be supported with cognitive behavioural approaches (Pontillo et al., 2019). This study suggests that particularly among children who have been severely bullied, beliefs associated with external locus of control may be relevant to maintaining and exacerbating social anxiety. More specific investigation of these beliefs could inform targeted and developmentally appropriate approaches to treatment among young people. In addition, these findings underline the importance of repeated measurement of social anxiety during adolescence in order to recognize differential trajectories of change. It appears that the differentiated trajectories in social anxiety were not visible prior to age 10 and rather became apparent first between age 10 and 13. This underscores the importance of developmental models of psychopathology that can be linked to a developmental clinical approach.

Some limitations in this study should be kept in mind. The sample suffered from attrition but this is common in prospective studies of this duration. Although higher exposure to bullying was associated with dropout, this would have if anything likely attenuated findings rather than increased them, such that effect sizes may have been greater if these participants had been retained. Small effect sizes indicate that substantial variance in the model remains unexplained, perhaps due to cognitive or social factors that were not measured, or due to biological or genetic factors. Observed effects between bullying and social anxiety accounted for early internalizing problems, but these were measured after starting school (age 6), so the possibility that very early bullying triggered anxiety cannot be ruled out. It is also possible that past or ongoing bullying may negatively impact self-esteem later, but this is not measured in this study. Of note, the locus of control and self-esteem measures used in this study were not developed within the cognitive model framework but can provide a useful proxy for the meaning of the constructs within this model. Future research should assess whether the observed effect of external locus of control on social anxiety is indeed replicated for cognitions that are consistent with this construct and tailored to perceptions of bullying experiences.

Overall, it is well known that bullying contributes to increased risk of anxiety among children. It is also known that this is a critical developmental stage for increasing social anxiety symptoms and onset of social anxiety disorder, a mental health problem with severe consequences which once chronic rarely abates in the absence of specific interventions. It is also widely recognized that cognitive factors are central to the onset and maintenance of anxiety disorders including social anxiety. The present study aimed to understand the impact of specific cognitive factors, namely locus of control and self-esteem, on trajectories of social anxiety among children aged 8 to 13. Results suggest that children who are severely bullied at age 8 are particularly at risk of increasing social anxiety if they also hold an external locus of control. However, self-esteem does not appear to have the same moderating effect. It is possible that beliefs consistent with external locus of control contribute to further reduced perception of control over the environment in the context of bullying, which leads to more passive or ineffective coping strategies. The results of this study offer new insight into potentially modifiable factors that increase risk of social anxiety among bullied children and suggest that external control beliefs could be useful targets for cognitive interventions.

## Data Availability

Conditions for accessing and using ALSPAC data are described on the study website http://www.bristol.ac.uk/alspac/researchers/access/
